# Automated Risk Assessment of Opioid Use: Analysis Using Pre-Trained Transformers on Social Media Data

**DOI:** 10.2196/77783

**Published:** 2026-02-19

**Authors:** Muhammad Ahmad, Rita Orji, Maaz Amjad, Abubakar Siddique, Nailya Kubysheva, Ildar Batyrshin, Grigori Sidorov

**Affiliations:** 1 Instituto Politécnico Nacional Centro de Investigación en Computación Mexico City Mexico; 2 Faculty of Computer Science Dalhousie University Halifax, NS Canada; 3 Department of Computer Science Texas Tech University Lubbock, TX United States; 4 School of Engineering, Computer and Mathematical Sciences Auckland University of Technology Auckland New Zealand; 5 Kazan Federal University Kazan Russian Federation

**Keywords:** opioid overdose, chronic pain, data mining, social media, deep learning, transformer, BERT, drug abuse, Reddit, AI, artificial intelligence

## Abstract

**Background:**

The illegal use of opioids has emerged as a major global public health concern, contributing to widespread addiction and a growing number of overdose-related deaths. In response, the US federal government has invested billions of dollars in combating the opioid epidemic through treatment, prevention, and law enforcement initiatives. Despite these efforts, there remains an urgent need for automated tools capable of detecting overdose cases and assessing the risk levels of substances—tools that can enable faster, more effective responses with less reliance on human intervention. Social media, particularly Reddit, has become a valuable source of self-reported data on opioid misuse, offering rich insights into user experiences and symptoms.

**Objective:**

This research aimed to develop an advanced automated tool for detecting opioid overdose risks and classifying substances into high-risk and low-risk categories by analyzing social media posts.

**Methods:**

A multistage methodology was used to achieve the objectives of this work. First, a new dataset was constructed from Reddit posts and manually annotated. Each post was labeled according to the risk level of the mentioned substance, using contextual indicators and user-reported experiences as the basis for classification. To ensure reliability and annotator consistency, detailed annotation guidelines were developed and applied throughout the labeling process. Second, a bidirectional encoder representation from transformers for biomedical text mining (BioBERT)–based classification framework was implemented and enhanced with a custom attention mechanism to capture relevant semantic information for more accurate predictions. Third, the model’s performance was evaluated using 5-fold cross-validation and compared against several baseline approaches, including traditional supervised learning, deep learning, and transfer learning methods. In total, 14 experiments were conducted to evaluate comparative effectiveness. To further assess the contribution of the attention layer, the best-performing model was also evaluated against a version incorporating the standard self-attention mechanism, using a train-test split. Finally, a paired *t* test was conducted to statistically assess the performance difference between the BioBERT-based model and the strongest baseline, extreme gradient boosting (XGBoost), providing validation of the observed improvements.

**Results:**

The proposed BioBERT model with custom attention achieved an *F*_1_-score of 0.99 in cross-validation, outperforming the best baseline, XGBoost (*F*_1_-score=0.97), with a relative improvement of 2.06%. A paired *t* test conducted across the 5 folds (n=5) confirmed that the performance gain was statistically significant (*P*=.003), providing strong evidence that the improvement reflects genuine advances in overdose risk detection.

**Conclusions:**

This paper demonstrates the potential of leveraging social media data and advanced natural language processing models to build reliable systems for opioid overdose risk detection. The BioBERT model with custom attention shows state-of-the-art performance and robustness, offering a powerful tool to support timely intervention and harm reduction strategies in the ongoing opioid crisis.

## Introduction

### Background

Chronic pain has become a major public health challenge worldwide, affecting more than 25 million adults in the United States [1,2], with prevalence rates ranging from 11% to 40% [3]. Treatment of chronic pain is a complex process, and most treatment advice proposes a combination of psychological and pharmacological methods [4-6], and pharmacological treatments include both prescribed and illicit medications [3].

Nonprescribed medications, such as paracetamol, codeine, tramadol, ibuprofen, and aspirin, are frequently used for mild pain management when medical consultation is unnecessary, and they are usually considered harmless and effective [7]. Paracetamol is commonly used to relieve mild pain and is considered safe when administered according to recommended guidelines. However, there has been a surge in reports showing its misuse [7,8]. A study observing nonprescribed drug usage, such as paracetamol, showed that 24% of adults admitted to exceeding the recommended dosage [9]. Another study found that many people do not read the pharmacological instructions, and more than 50% do not know the active ingredient in the medication that they are taking [10]. Codeine and tramadol, classified as weak opioids, can sometimes be obtained as nonprescribed drugs, despite their potential for side effects [11].

Meanwhile, the misuse of prescription opioids and illicit drugs, such as heroin and fentanyl, has resulted in a record number of opioid-related overdoses and fatalities. According to the Centers for Disease Control and Prevention (CDC), opioids were involved in approximately 70% of all drug overdose deaths in the United States [12]. This crisis presents significant health risks [13], strains health care systems, contributes to social unrest, and intensifies economic challenges. Addressing this issue requires multifaceted strategies that go beyond traditional clinical settings, incorporating preventive measures and rapid responses to emerging risks.

Moreover, despite being illegal, the online sale of prescription medications has been documented [11,14]. Twitter (now known as X; developed by X Corp) has emerged as a platform where illegal transactions involving these drugs occur. Sales on Twitter are often less regulated and monitored compared to those on other online platforms [15]. Similarly, Reddit has also been identified as a place where individuals engage in discussions or transactions related to prescription drugs, often with minimal oversight [16,17].

Social media is an important source of user-generated content that offers valuable insights into opioid misuse. Reddit is a popular social media platform and is ranked as the 9th-largest social media platform in the United States [18]. It provides enough space to express self-reported experiences, with a maximum character limit for a text post of 40,000 characters. These discussions offer a unique opportunity to better understand the hidden patterns and risks associated with opioid misuse, which often go overlooked in clinical settings.

In recent years, natural language processing (NLP) has arisen as a powerful tool for addressing and understanding the opioid crisis through social media discourse. Nowadays, social media platforms such as X (formerly Twitter), Facebook (Meta Platforms, Inc), Reddit (Reddit, Inc), YouTube (Google LLC), and Instagram (Meta Platforms, Inc) have become real-time sources of information where people openly discuss their experiences, share their views, and behaviors related to opioid use. These platforms comprise a lot of textual, image, and video data that can be analyzed for sentiment analysis [19-22], named entity recognition [23-25], topic modeling [21,26], text classification [27,28], and opioid-related risks, such as overdose incidents, patterns of misuse, and emerging trends in substance abuse [29,30]. Through advanced NLP methods, it is possible to automatically categorize and evaluate the risk levels of posts related to opioids, providing a timely and scalable method to identify individuals at risk and inform public health interventions.

Unlike traditional clinical settings, social media provides a unique outlet where individuals can candidly share their experiences with opioid use, often motivated by anonymity, community support, and reduced stigma. These self-reported narratives offer critical, real-time insights into opioid misuse patterns that may go unreported in clinical data. While prior studies have used NLP to analyze social media content, most rely on unsupervised methods (eg, topic modeling and sentiment analysis) or weakly labeled data. These approaches often lack the granularity and clinical validity needed for precise risk assessment. Whereas, advanced transformer-based models applied in earlier studies typically do not incorporate domain-specific annotation schemes, limiting their effectiveness in identifying high-risk content.

To address these limitations, this work aims to manually annotate a new dataset using expert-informed risk guidelines, enabling supervised training of models to directly classify opioid-related posts as high-risk or low-risk. Moreover, this study integrates recent advances in NLP with clinically grounded annotation and empirical benchmarking across both traditional and deep learning models, including transformers. This approach supports the development of a scalable system for real-time detection of opioid misuse risk on social media, facilitating timely public health interventions.

To further improve the prediction accuracy of deep learning models, this work introduces a novel architectural enhancement by integrating a custom attention mechanism in bidirectional encoder representations from transformers for biomedical text mining (BioBERT)–based contextualized representations. The attention layer dynamically assigns weights to token embeddings, enabling the model to focus more effectively on the most informative parts of the input text. Such targeted representation learning is expected to enhance the model’s ability to capture nuanced patterns associated with opioid misuse. It is hypothesized that this strategy will play a key role in achieving superior empirical performance in detecting high-risk opioid substance use.

This study makes the following contributions: (1) the creation of a manually annotated dataset specifically designed to classify opioid substance use into high-risk and low-risk categories. The corpus is annotated by identifying key indicators and symptoms from social media posts; (2) the development of comprehensive annotation guidelines in collaboration with domain experts to ensure consistent and accurate identification of misuse patterns. These guidelines support early intervention efforts and can inform public health strategies for risk reduction and timely response; and (3) the introduction of a novel architectural enhancement inspired by transformer models, in which a custom attention mechanism is integrated into BioBERT’s contextualized representations. This approach is expected to improve the model’s ability to capture subtle, high-risk patterns associated with opioid misuse.

### Prior Work

This section provides an overview and critical analysis of existing studies on substance use monitoring, opioid-related discourse, and public health surveillance.

#### Social Media Analysis for Substance Use Monitoring

Several studies have used NLP techniques to monitor drug-related discourse on social media. For instance, Garg et al [17] manually annotated Reddit posts related to fentanyl using a clinically informed codebook comprising 12 risk categories. Subsequently, they trained machine learning models to classify risky content, achieving 76% accuracy and sensitivity. Their work also uncovered slang terms for fentanyl and its analogues, aiding early risk detection. However, their focus remained limited to a single substance and a binary risk classification.

Similarly, Dunn et al [31] conducted a thematic analysis of Reddit discussions on veterinary drug misuse, particularly xylazine and carfentanil. Their qualitative and AI-assisted approach highlighted the potential misuse, motivations, and adverse effects. While valuable for trend detection, their approach lacks automated, supervised classification. In contrast, this work broadens the scope across multiple opioids and introduces a supervised binary classification (high risk vs low risk) using a manually annotated dataset, enhancing the generalizability and automation of risk detection.

Jha et al [32] developed a computational method to identify and characterize the stages of opioid addiction using users’ social media posts. They combined recurrent neural networks, information-theoretic word association analysis, and context-based word embeddings to detect addiction stage–specific language patterns. To identify users at high risk of relapse, they applied propensity score matching and logistic regression techniques. Their approach demonstrated high accuracy in distinguishing addiction stages and relapse risk, achieving *F*_1_-scores of 0.88 and 0.79, respectively.

Smith et al [33] investigated the potential of Reddit as a real-time data source for monitoring the US opioid epidemic, focusing on heroin, prescription, and synthetic opioids. They developed an NLP-based pipeline to identify opioid-related content. Moreover, they created a large user cohort of over 1.6 million Reddit users, assigning each to a US state. By tracking opioid-related posts over time, they compared Reddit-based trends with CDC overdose data and National Forensic Laboratory Information System drug reports. Incorporating Reddit data into overdose prediction models significantly improved accuracy, highlighting the value of social media for timely public health surveillance.

#### Sentiment and Emotion Analysis in Opioid-Related Discourse

Emotion and sentiment play a central role in substance use discussions. Gandy et al [34] evaluated multiple sentiment analysis tools—including VADER, LIWC-22, and ChatGPT 4.0—on YouTube comments about the opioid epidemic. While VADER performed best for identifying negative sentiment, and LIWC-22 for prevalence estimation, their study did not support real-time prediction or classification of risk. Yang et al. [35] used a GAN-based model to predict opioid relapse through sentiment features extracted from Reddit posts. By converting emotional content into “sentiment images,” their model captured relapse indicators such as “joy” and “negative” emotions effectively. However, they aimed at predicting relapse, not general risk detection. Similarly, Lokala et al [24] applied deep learning to detect substance use disorder discussions, correlating emotions such as withdrawal and addiction with synthetic opioid mentions. Although their work achieved strong performance (*F*_1_-score=82.12), it emphasized emotion tracking rather than comprehensive risk classification. In contrast, this study integrates an explainable transformer-based model (BioBERT with custom attention) to move beyond emotion detection toward direct, interpretable risk classification of opioid-related content.

#### Topic Modeling and Public Health Surveillance

Li et al [36] developed a comprehensive pipeline for analyzing COVID-19 drug discourse on Twitter using named entity recognition, sentiment analysis, topic modeling, and drug network mapping. Although their framework processed an extensive dataset of 169 million tweets, it lacked ground-truth annotations and supervised classification, thereby constraining its predictive and validation capabilities.

In another study, Zhang et al [37] examined Reddit posts to understand how teens frame substance use, revealing 7 social-emotional themes such as normalization, coping, and stigmatization. Their focus on emotional framing and qualitative analysis is insightful but not predictive. The novel system proposed here fills these gaps by offering a ground-truth–labeled, supervised classification system capable of delivering real-time, interpretable predictions. This approach enhances both the predictive validity and practical applicability of social media analytics for public health monitoring and early intervention.

While prior studies have made significant contributions across thematic analysis, sentiment tracking, and topic modeling, they often lack supervised classification or are constrained to a single substance. Building upon these foundations, this work proposes a generalizable, supervised deep learning framework for real-time, risk-level classification of opioid-related Reddit posts, empowered by expert annotations and explainable attention mechanisms.

## Methods

### Dataset Construction and Preprocessing

To systematically organize high-risk and low-risk opioid substances, a comprehensive list of relevant keywords was compiled. It includes substance names as well as general terms related to opioid use. This strategy helped to ensure broad coverage and improved generalization in data collection. The complete list of keywords is provided in Table 1.

**Table 1:** Keywords used to extract the dataset.

Category	Keywords used
High-risk substance use	overdose, fentanyl, heroin, oxycodone, opioid abuse, withdrawal, painkillers, substance dependence, addiction, relapse, injecting opioids, drug misuse, narcotics, high dosage, opioid crisis
Low-risk substance use	pain management, prescribed medication, controlled dosage, opioid awareness, recovery, tapering off, medical prescription, safe use, harm reduction, medication-assisted treatment (MAT), doctor-recommended, responsible use
General keywords	opioid, pain relief, medication, painkillers, chronic pain, prescription drugs, opioid discussion, rehab, opioid safety, self-medication

A Python-based data collection pipeline was developed using a keyword dictionary to extract Reddit posts via the Pushshift.io application programming interface (developed by Jason Baumgartner). Covering the period from January 1, 2022, to August 8, 2024, a total of 30,000 posts were gathered from several opioid-related subreddits, including r/opiates [38] and r/OpiatesRecovery [39], r/heroine [40], and r/addiction [41]. The posts were divided into 2 categories, namely, high-risk opioid use (eg, overdose cases and addiction symptoms) and low-risk opioid use (eg, responsible medication usage and recovery experiences). Moreover, each post was manually labeled either as high-risk or low-risk opioid use. Consequently, the dataset provides a strong foundation for training machine learning models to identify risk patterns in opioid-related social media discourse and to improve public health interventions.

**Figure 1:**
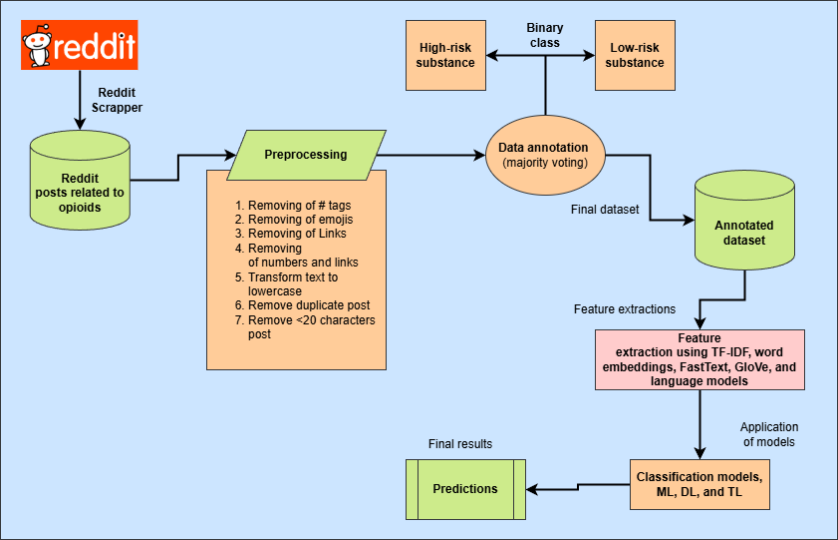
Proposed architecture and design. DL: deep learning; GloVe: Global Vectors for Word Representation; ML: machine learning; TL: transfer learning; TF-IDF: term frequency–inverse document frequency.

A comprehensive text preprocessing pipeline was created to standardize the content and minimize noise in the collected posts. It converted all text to lowercase, expanded contractions, and translated common abbreviations using a custom-built dictionary specifically adapted for adolescent languages (eg, converting “idk” to “I do not know”). Moreover, it removed all the HTML tags, numeric values, punctuation marks, and special characters. Furthermore, it normalized elongated or repeated characters in words (eg, transforming “loooove” into “love”), filtered standard and domain-specific stop words using an extended version of the Natural Language Toolkit. English stop word list, and applied lemmatization to reduce words to their root forms. Duplicate entries were eliminated based on the cleaned content. Posts with fewer than a specific number of characters (eg, 20) were discarded to maintain the quality and relevance of the dataset. Finally, posts irrelevant to the selected topic or written in languages other than the specified ones were removed manually. Consequently, a cleaned dataset consisting of 4739 posts was prepared for analysis. Figure 1 illustrates the overall architecture and methodology of the proposed approach.

### Annotation

Annotation is the process of assigning a predefined label to each sample to create a structured dataset that can be used to train a machine learning model. In opioid substance classification, annotation plays an important role in correctly categorizing each sample as either high-risk or low-risk based on its content.

### Annotation Guidelines

To ensure consistency and reliability in the annotation process, the following 13 detailed annotation guidelines were developed to support the binary classification of Reddit posts:

1. Explicit mentions of high-risk substances: read each sample carefully and check whether the user consumes substances such as heroin, fentanyl, oxycodone, morphine, methadone, or other strong opioids that are labeled as high-risk.
2. Explicit mentions of low-risk substances: read carefully and check whether the social media posts mention medications such as paracetamol, ibuprofen, aspirin, tramadol (in moderate doses), or nonopioid pain relievers that are labeled as low-risk.
3. Overdose mentions: posts describing overdose symptoms, emergencies, or near-fatal incidents are categorized as high-risk.
4. Illegal drug transactions: if a user mentions buying, selling, or obtaining opioids illegally (especially from online sources), the post is labeled as high-risk.
5. Prescription use vs misuse: if any posts referring to prescription adherence are labeled as low-risk, whereas discussions about taking more than the prescribed dose are considered high-risk.
6. Polysubstance use: if a post mentions mixing opioids with alcohol, benzodiazepines, or other sedatives, it is classified as high-risk due to increased overdose potential.
7. Self-medication for pain management: if a user in the post mentions self-medicating with low-risk pain relievers such as ibuprofen, aspirin, or paracetamol, it is classified as low-risk.
8. Withdrawal symptoms and addiction struggles: if any user in the posts reports withdrawal symptoms, cravings, or dependence on opioids, the post is labeled as high-risk.
9. Recreational use: if any user in the post explicitly mentions opioid use for recreational purposes or to get high, it is categorized as high-risk.
10. Seeking help or support: if the post seeks medical advice, rehabilitation, or addiction recovery support, it is labeled based on the context—if high-risk substances are mentioned, it is high-risk; otherwise, it is labeled as low-risk.
11. Medical advice discussions: if users discuss doctor-prescribed medications for pain management, the post is classified as low-risk unless signs of misuse are present.
12. Harm reduction strategies: if a post provides information on safe opioid use, naloxone administration, or overdose prevention, it is categorized as low-risk.
13. Slang and codewords: posts containing slang terms for high-risk opioids (eg, “H” for heroin and “china white” for fentanyl) are considered high-risk, while those referring to nonopioid alternatives remain low-risk.

Table 2 provides representative examples of Reddit posts categorized as high-risk and low-risk substance use based on the above guidelines. These examples illustrate the distinctions in language patterns, context, and behavioral cues that guided the annotation process.

**Table 2:** Examples of high-risk and low-risk substance use posts taken from the dataset.

Post	Risk level
The first time I tried heroin, I took a high dose, and it was the worst experience of my life. I felt an intense rush, but then everything went dark. I couldn’t think straight, and I felt like I was going to pass out. It scared me how quickly I lost control, and I swore I’d never touch it again. It's a dangerous substance, and I regret ever trying it.	High risk
I was prescribed oxycodone after surgery, and at first, it helped with the pain. But when I took a higher dose for pain management, it made me feel dizzy and out of it, like I wasn’t fully there. It was too much, and I started to realize how easily you can lose control.	High risk
I’ve been using kratom for a while to help with chronic pain, as recommended by my doctor. The first time I tried it, it gave me a mild sense of relief—like a calm energy boost, without any highs or intense effects. It’s been a good alternative for me, and I can take it without feeling out of control. My doctor keeps track of my usage, and it feels safer than other options.	Low risk
I’ve been using paracetamol for years to manage minor aches and pains, like headaches or body aches. It’s effective and works fast, without making me feel drowsy or out of it. I take it as directed by my doctor and never exceed the recommended dose, as it’s important to avoid liver damage. It’s a safe, low-risk option for occasional pain relief.	Low risk

### Annotation Selection

To ensure high-quality annotations for the dataset, a 2-stage selection process was devised to identify the most reliable annotators. In the first round, 500 Reddit posts were provided to 8 annotators to label the posts based on the above-mentioned annotation guidelines. After evaluating their responses, it was found that only 5 annotators assigned nearly identical labels, indicating consistency in their understanding of the task. In the second round, another set of 500 posts was assigned to the 5 annotators who showed consistency in their understanding. Their responses were again evaluated; only 3 annotators demonstrated high-quality labeling with strong agreement, making them the most reliable for dataset construction. To track annotator performance, Google Forms were used for individual assessments. Google Forms played a critical role in (1) standardizing the annotation process, ensuring all annotators follow the same guidelines; (2) collecting and analyzing responses, identifying discrepancies in labeling; and (3) monitoring consistency over time, assisting in selecting the most reliable annotators. The final 3 annotators were master’s students with strong backgrounds in machine learning and NLP. While they were not clinical or addiction medicine professionals, the annotation guidelines were developed in consultation with domain experts to ensure clinical relevance. Annotators received training based on these guidelines, and ambiguous cases were resolved through collaborative discussion and expert input when necessary. To address any uncertainties or disagreements in annotation, we scheduled weekly meetings to discuss and resolve ambiguities by majority voting, as provided in Figure 1. Through this rigorous selection and validation process, it was ensured that the dataset was robust, accurate, and reliable for further processing and model training.

### Interannotator Agreement

Interannotator agreement is a process used to measure the consistency and reliability of annotations between different annotators. It supports the assessment of whether the annotations are reliable or whether there are inconsistencies that need to be resolved. In this paper, Cohen κ was calculated to evaluate the agreement between annotators for classifying Reddit samples into a binary class. In this study, the Cohen κ value was 0.79, which shows a substantial agreement between the annotators, as shown in Table 3. This level of agreement is sufficient for ensuring that the annotations are consistent and reliable for further analysis and model training.

**Table 3:** Interpretation of the κ values.

Cohen κ value range	Interpretation
1.0	Perfect agreement
0.80-1.00	Substantial agreement
0.60-0.80	Moderate agreement
0.40-0.60	Fair agreement
<0.40	Poor agreement

### Dataset Statistics

A comprehensive statistical analysis was performed to gain a deeper understanding of the dataset’s composition and linguistic characteristics. Multimedia Appendix 1 provides a word cloud comprising keywords extracted from posts in the dataset related to the topic of opioid use and overdose. The word cloud visually highlights the most frequent terms, emphasizing the critical themes discussed in the dataset. Multimedia Appendix 2 provides the distribution of labels for each class used in the corpus for sentiment analysis. The chart visually represents the frequency of each sentiment class in the dataset. Multimedia Appendix 3 provides dataset statistics related to high-risk or low-risk substance use. It includes 4739 posts, totaling 344,827 words and 1,567,169 characters, with a vocabulary of 14,905 unique words. On average, each sentence contains 16.65 words, and each post consists of about 4.37 sentences. The average word length is 4.54 characters, and each post typically contains 330.7 characters. These metrics help analyze language complexity, engagement, and potential differences in how individuals discuss substance use risk contexts.

### Feature Extraction

To extract relevant and valuable features for opioid overdose risk detection, a combination of traditional and modern feature extraction techniques was used: term frequency–inverse document frequency (TF-IDF), pretrained word embeddings (FastText and Global Vectors for Word Representation [GloVe]), and contextualized embeddings derived from transformer-based language models. The TF-IDF approach represents textual data by weighing each word based on its frequency in a document and its rarity across the entire corpus. This technique effectively downplays common but uninformative words while emphasizing more meaningful terms for classification. TF-IDF consists of 2 components, term frequency (TF) and inverse document frequency (IDF). The TF measures the relative frequency of a word with respect to the total number of words in the document:

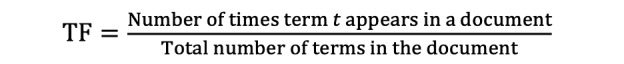



The IDF of a term reflects the inverse proportion of documents containing that term. Terms with technical jargon hold greater significance than words found in only a small percentage of all documents. The IDF is calculated using the following equation:

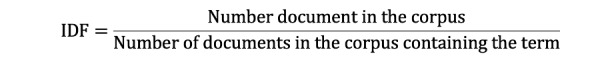



Finally, the TF-IDF score for each term is obtained by multiplying its TF and IDF values, as shown in equation 3:

TF – IDF = TF × IDF (3)

This representation enhances the ability of machine learning models to capture the most informative linguistic patterns associated with opioid-related risk discourse.

Two dense vector representations based on pretrained word embeddings are used: FastText and GloVe. These techniques provide fixed-dimensional representations while capturing semantic and syntactic properties. The FastText technique represents words as bags of character n-grams, enabling better handling of out-of-vocabulary or misspelled words common in noisy social media text. Specifically, it extends Word2Vec by representing words as bags of character n-grams. The embedding for a word “w” is calculated as follows:

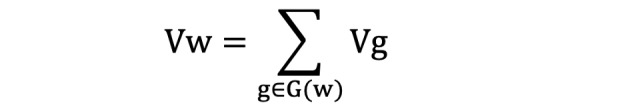

- G (w) is the set of character n-grams in the word w.
- Vg is the vector representation of each n-gram g.

The GloVe generate word embedding based on the global word co-occurrence matrix of words, effectively modeling semantic relationships. The cost value for GloVe is computed using the ratio of co-occurrence probabilities:

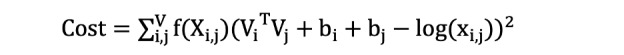



Where:

- Xi,j​ is the number of times word j occurs in the context of word i.
- V is the vocabulary size.
- Vi and Vj are the embeddings for words i and j.
- bi and bj​ are bias terms for the words.
- f(Xi,j) is a weighting function to down-weight the influence of very frequent words.

The FastText and GloVe techniques are selected for study due to their complementary strengths: FastText addresses linguistic variations and out-of-vocabulary words in Reddit discussions on opioid use, while GloVe captures broader semantic relationships across the corpus. These embeddings provide richer representations than sparse features and were used to train deep learning architectures such as a convolutional neural network (CNN) and a bidirectional long short-term memory (BiLSTM). It is noted that Word2Vec is not used because it lacks subword-level understanding and relies mainly on local context windows, making it less robust for the lexical noise present in a dataset.

To further enhance contextual understanding, transformer-based language models are used to extract contextualized embeddings. These models generate dynamic representations of words based on their surrounding context, addressing challenges such as polysemy, ambiguous expressions, and informal language.

### Application of Machine Learning Models

**Figure 2:**
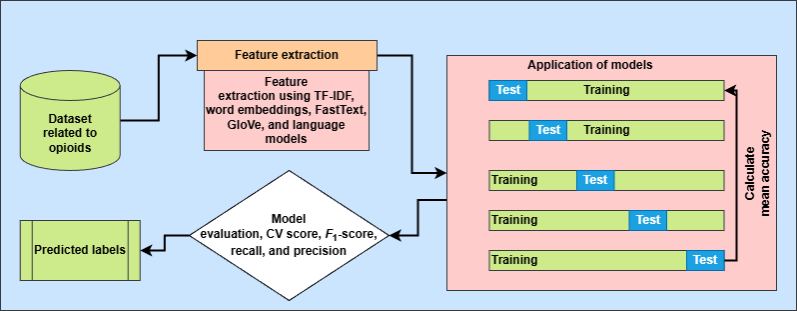
Application of machine learning models. CV: cross-validation; GloVe: Global Vectors for Word Representation; TF-IDF: term frequency–inverse document frequency.

A diverse set of machine learning models is applied to the opioid dataset for thorough analysis and accurate classification of social media posts (Figure 2). This includes transformer-based architectures with a custom attention mechanism, such as BioBERT, Robustly Optimized BERT Pretraining Approach, XLM-RoBERTa (Cross-lingual Language Model–Robustly Optimized BERT Pretraining Approach), and ELECTRA (Efficiently Learning an Encoder that Classifies Token Replacements Accurately), traditional machine learning models (decision tree [DT], extreme Gradient Boosting [XGB], multinomial naive Bayes [MNB], and adaptive boosting [AdaBoost]), and commonly used deep learning models (BiLSTM and CNN). Each model in the set captures different aspects of the analysis and classification tasks. For example, DT offers interpretability and handles nonlinear decision boundaries; XGB and AdaBoost provide ensemble-based robustness for high-dimensional features; MNB serves as an efficient probabilistic baseline; CNN captures local n-gram patterns, while BiLSTM models long-range dependencies in both directions.

The contribution of this work lies in a methodologically rigorous evaluation pipeline for assessing opioid risk levels in social media posts, integrating multiple learning paradigms and focusing on real-world Reddit data. Central to this approach is a fine-tuned BioBERT model with a custom attention mechanism, capturing nuanced linguistic and contextual cues.

To enhance the transformer models’ ability to capture task-specific linguistic cues in opioid-related discourse, a custom attention layer is used on top of the transformer’s contextualized representations, which—unlike standard self-attention—assigns greater weights to domain-relevant tokens (eg, “OD,” “relapsed,” “detox,” and “pill count”) through a learnable scoring function. This enables the model to emphasize medically significant phrases, emotional expressions, and slang terms, ultimately improving both the interpretability and precision of risk-level predictions.

Table 4 provides the hyperparameters and their grid search values across models, demonstrating careful attention to optimization and reproducibility. For transformer-based models, the custom attention mechanism dynamically weighs token embeddings. Hyperparameters include a learning rate of 3 × 10⁻⁵, 3 epochs, batch size 16, AdamW optimizer, and CrossEntropyLoss for multiclass classification. The attention layer consists of 2 linear layers (768 → 512 → 1) with a Tanh activation, introducing nonlinearity and enabling token-level focus.

**Table 4:** Optimum values for the hyperparameters of the proposed models.

Learning approach and models			Hyperparameters		Grid search values
**Transformer**					
	BERT^a^, RoBERTa^b^, XLM-RoBERTa^c^, and ELECTRA^d^ (with custom attention)	Learning rate, epochs, batch size, optimizer, and loss function		3 × 10⁻⁵, 3, 16, AdamW, and CrossEntropyLoss	
**Machine learning**					
	XGB^e^	n_estimators, max_depth, and learning_rate		100, 6, and 0.3	
	MNB^f^	Alpha (smoothing parameter) and fit prior (fit_prior)		alpha: 0.1, 0.5, 1.0 and fit_prior: true, false	
	DT^g^	Maximum depth of the tree (max_depth), minimum samples for splitting (min_samples_split), minimum samples for leaf nodes (min_samples_leaf), and maximum features for splitting (max_features)		max_depth: 5, 10, 20, and none; min_samples_split: 2, 5, and 10; min_samples_leaf: 1, 2, and 4; and max_features: “sqrt,” “log2,” and none	
	AdaBoost^h^	Number of estimators (n_estimators), learning rate (learning_rate), and base estimator (base_estimator)		n_estimators: 50, 100, and 200; learning_rate: 0.01, 0.1, and 1.0; base_estimator: DecisionTreeClassifier(max_depth=1)	
**Deep learning**					
	BiLSTM^i^	Learning rate, epochs, Embedding_dim, batch size, and LSTM^j^ units		0.1, 5, 300, 32, and 128	
	CNN^k^	Learning rate, epochs, Embedding_dim, batch size, filters, and kernel size		0.1, 5, 300, 32, 128, and 5	

^a^BERT: Bidirectional Encoder Representations from Transformers.

^b^RoBERTa: Robustly Optimized BERT Pretraining Approach.

^c^XLM-RoBERTa: Cross-lingual Language Model–Robustly Optimized BERT Pretraining Approach.

^d^ELECTRA: Efficiently Learning an Encoder that Classifies Token Replacements Accurately.

^e^XGB: extreme Gradient Boosting.

^f^MNB: multinomial naive Bayes.

^g^DT: decision tree.

^h^AdaBoost: adaptive boosting.

^i^BiLSTM: bidirectional long short-term memory.

^j^LSTM: long short-term memory.

^k^CNN: convolutional neural network.

Traditional machine learning models are tuned as follows: XGB used 100 estimators, a max depth of 6, and a learning rate of 0.3; MNB used alpha values from 0.1 to 1.0 and fit_prior was set to true or false; DT explored max_depth (5, 10, 20, and none), min_samples_split (2, 5, and 10), and other parameters governing tree complexity; AdaBoost used n_estimators (50, 100, and 200), learning_rate (0.01, 0.1, and 1.0), and a base DT with max_depth 1.

Deep learning models are optimized with BiLSTM using a learning rate of 0.1, 5 epochs, an embedding dimension of 300, a batch size of 32, and 128 long short-term memory units. CNN has similar hyperparameters with 128 filters and a kernel size of 5. These settings are selected to balance learning capacity, computational efficiency, and model performance, optimized through grid search on validation data.

To assess generalization, the proposed model is trained on posts from 2022 to 2023 and evaluated on posts from 2024. The 5-fold cross-validation (CV) and paired *t* tests are applied to ensure replicable and statistically robust performance. This strategy bridges domain-specific insights with advanced computational techniques, yielding results relevant for public health surveillance and intervention strategies.

### Ethical Considerations

This study was conducted in accordance with established ethical standards for research involving online data. The dataset was compiled exclusively from publicly accessible social media platforms. “Publicly available” refers to content that can be accessed freely by any internet user without login requirements, private group membership, or special permissions.

As the research relied solely on publicly available data and did not involve interaction with human participants, intervention, or access to private or restricted information, formal Institutional Review Board (IRB) approval was not required under standard ethical guidelines governing research on publicly accessible online content.

To ensure the protection of user privacy and minimize the risk of re-identification, rigorous safeguards were implemented. No personally identifiable information was collected or stored. All direct identifiers (eg, usernames, profile details, account IDs) were removed during data preprocessing, and the dataset was fully anonymized prior to analysis. Verbatim quotes were avoided or paraphrased to prevent traceability through search engines, and any contextual details that could potentially enable identification were excluded. All analyses were conducted and reported in aggregate form.

These procedures were undertaken to ensure responsible, privacy-conscious, and trustworthy use of publicly available online data.

## Results

### Traditional Machine Learning Results

This section provides the experimental results of the machine learning models applied to opioid risk detection using social mdia data. The traditional machine learning models, such as DT, XGB, AdaBoost, and MNB, are evaluated based on their performance in classifying high-risk and low-risk opioid-related posts.

Table 5 provides the results of 4 different machine learning models—XGB, MNB, DT, and AdaBoost—on a binary classification task to identify the opioid use risk as either high or low. The performance of each model is evaluated based on 4 standard evaluation metrics, that is, precision, recall, *F*_1_-score, and CV score. Across all metrics, XGB outperforms all other models with a CV score of 0.97, showing its higher capability in distinguishing between high-risk and low-risk cases. DT follows with scores ranging from 0.94 to 0.95, indicating solid but slightly lower performance. AdaBoost also achieves comparable performance, maintaining steady scores of 0.94 across all metrics. On the other hand, MNB shows the weakest performance, especially in recall and *F*_1_-score (0.93), showing limitations in capturing the nuances of the classification problem. Overall, XGB appears to be the best-performing model for this task in identifying opioid use risk.

**Table 5:** Results for machine learning models.

Metric	XGB^a^	MNB^b^	DT^c^	AdaBoost^d^
Precision	0.97	0.94	0.95	0.94
Recall	0.97	0.93	0.95	0.94
*F*_1_-score	0.97	0.93	0.95	0.94
CV^e^ score	0.97	0.93	0.95	0.94

^a^XGB: extreme Gradient Boosting.

^b^MNB: multinomial naive Bayes.

^c^DT: decision tree.

^d^AdaBoost: adaptive boosting.

^e^CV: cross-validation.

### Deep Learning Model Results

This section provides the experimental results of the 2 commonly used deep learning models, namely, BiLSTM and CNN. By leveraging the power of deep learning, these models are used to capture more complex patterns in the data and improve the detection of opioid misuse and overdose risks.

Table 6 compares the experimental results of deep learning models—CNN and BiLSTM—using 2 different word embeddings: FastText and GloVe, for the same binary classification task of identifying high- vs low-risk of opioid use. With FastText embeddings, both CNN and BiLSTM perform well, with BiLSTM slightly outperforming CNN across all metrics (precision, recall, *F*_1_-score, and CV score) at 0.94 compared to CNN’s consistent 0.92. When using GloVe embeddings, CNN demonstrates even stronger performance, achieving 0.95 across all metrics, making it the best-performing model among the deep learning approaches evaluated. However, BiLSTM with GloVe embeddings underperforms significantly, especially in precision (0.83) and *F*_1_-score (0.85), suggesting that GloVe may not complement BiLSTM as effectively as FastText does for this specific task. Overall, the GloVe + CNN combination provides the highest performance, while the FastText + BiLSTM combination offers a strong alternative, indicating the critical influence of embedding choice on model effectiveness in opioid use risk classification.

**Table 6:** Results for deep learning models.

Models		Precision	Recall	*F*_1_-score	CV^a^ score
**FastText**					
	CNN^b^	0.92	0.92	0.92	0.92
	BiLSTM^c^	0.94	0.94	0.94	0.94
**GloVe** ^d^					
	CNN	0.95	0.95	0.95	0.95
	BiLSTM	0.83	0.88	0.85	0.88

^a^CV: cross-validation.

^b^CNN: convolutional neural network.

^c^BiLSTM: bidirectional long short-term memory.

^d^GloVe: Global Vectors for Word Representation.

### Transformer Model Results

This section provides the results of applying transformer-based models to the opioid risk detection task. These models are selected based on their superior performance in preliminary experiments and their widespread success in text classification tasks.

Table 7 provides the performance of transformer-based models—ELECTRA-base-discriminator, BioBERT, RoBERTa-base, and XLM-RoBERTa—each enhanced with a custom attention mechanism tailored for the binary classification of opioid use risk (high vs low). Among these, BioBERT demonstrates the highest performance, achieving near-perfect scores of 0.99 in precision, recall, *F*_1_-score, and CV score, underscoring the synergy between domain-specific pretraining and task-specific attention modeling. The remaining models—ELECTRA, Robustly Optimized BERT Pretraining Approach, and XLM-RoBERTa—also perform robustly, each scoring 0.98 across all metrics, indicating the effectiveness of the custom attention mechanism in improving general transformer architectures for the health-related NLP task.

**Table 7:** Transformer results.

Model		Precision	Recall	*F*_1_-score	CV^a^ score
electra^b^-base-discriminator		0.98	0.98	0.98	0.98
biobert^c^-base-cased-v1.1		0.99	0.99	0.99	0.99
roberta^d^-base		0.98	0.98	0.98	0.98
xlm^e^-roberta		0.98	0.98	0.98	0.98
**Train test split**					
	biobert-base-cased-v1.1	0.99	0.99	0.99	0.99
**Without a custom attention mechanism**					
	biobert-base-cased-v1.1	0.96	0.96	0.96	0.96

^a^CV: cross-validation.

^b^ELECTRA: Efficiently Learning an Encoder that Classifies Token Replacements Accurately.

^c^BioBERT: bidirectional encoder representations from transformers for biomedical text mining.

^d^RoBERTa: Robustly Optimized BERT Pretraining Approach.

^e^XLM-RoBERTa: Cross-lingual Language Model–Robustly Optimized BERT Pretraining Approach.

To evaluate the model on unseen data, the top-performing model is trained on posts from 2022 to 2023, saved, and then tested on a held-out set of approximately 948 posts from 2024 to assess its generalization performance. BioBERT maintained a precision, recall, and *F*_1_-score of 0.99 in this temporal evaluation, demonstrating that the model generalizes effectively to future, unseen data. These results indicate that the model’s performance is robust and not an artifact of overfitting, confirming its reliability for real-world applications. Additionally, these results confirm that while transformer models are inherently powerful, incorporating a custom attention mechanism further refines their focus on risk-relevant textual cues, especially when combined with a domain-adapted model such as BioBERT.

It is noted that the proposed model without the custom attention mechanism achieved 0.96 across accuracy, precision, recall, and *F*_1_-score, showing that it already captures much of the context in opioid-related text. Overall, by incorporating the custom attention layer, the performance improved to 0.99 on all metrics, demonstrating that emphasizing domain-specific tokens and phrases allows the model to make more precise and interpretable risk-level predictions.

### Error Analysis

Table 8, titled “top-performing models in each learning approach,” compares the highest-performing models from 3 distinct machine learning paradigms—XGB from traditional machine learning, CNN with GloVe embeddings from deep learning, and BioBERT model enhanced with custom attention mechanisms from transformer-based NLP—for classifying high- and low-risk opioid substances. The performance is measured across 4 metrics: precision, recall, *F*_1_-score, and CV score. The BioBERT model leads with a perfect score of 0.99 in all categories, showcasing its exceptional ability to capture deep semantic relationships within the data. XGB follows with solid performance, achieving 0.97 across the board, indicating that it remains a competitive option despite being a nondeep learning model. CNN (GloVe), while effective, ranks third with consistent scores of 0.95, suggesting that although it leverages word embeddings, it may lack the contextual understanding of transformers. This comparison clearly highlights the dominance of transformer models, such as Bidirectional Encoder Representations from Transformers, in handling complex, text-based substance risk classification tasks.

**Table 8:** Top-performing models in each learning approach.

Metric	XGB^a^	CNN^b^ (GloVe^c^)	BioBERT^d^
Precision	0.97	0.95	0.99
Recall	0.97	0.95	0.99
*F*_1_-score	0.97	0.95	0.99
CV^e^ score	0.97	0.95	0.99

^a^XGB: **e**xtreme Gradient Boosting.

^b^CNN: convolutional neural network.

^c^GloVe: Global Vectors for Word Representation.

^d^BioBERT: bidirectional encoder representations from transformers for biomedical text mining.

^e^CV: cross-validation.

The superior performance of the BioBERT model can be attributed not only to its bidirectional transformer architecture, which effectively captures deep contextual relationships in text data compared to traditional and shallow models such as XGB and CNN, but also to the integration of custom attention mechanisms that enable the model to focus on the most relevant parts of the input. This enhancement improves the detection of subtle, domain-specific patterns crucial for opioid risk classification.

**Figure 3:**
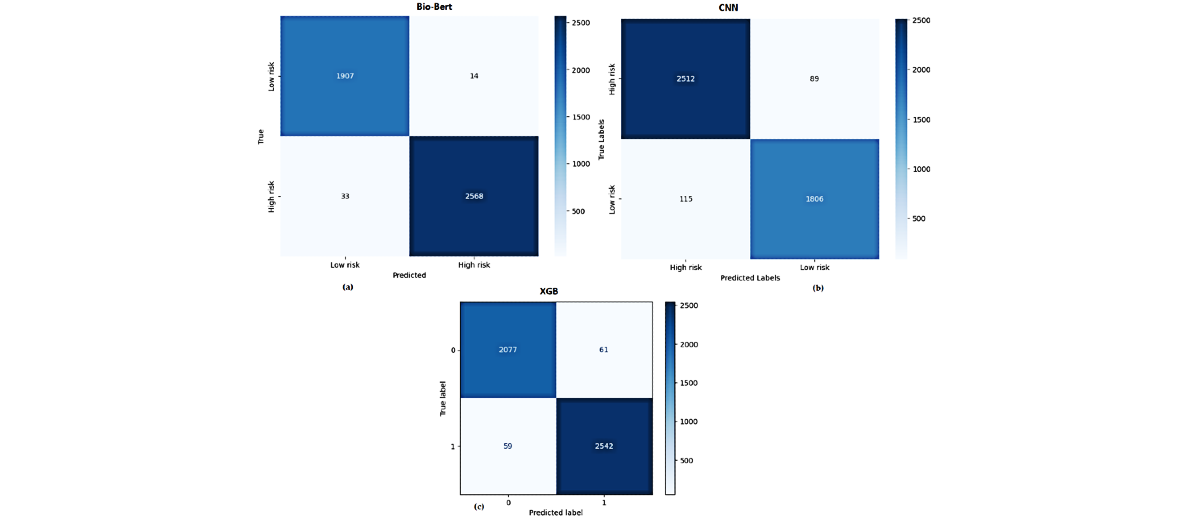
Confusion matrix of top-performing models in each learning approach. BioBERT: bidirectional encoder representations from transformers for biomedical text mining; CNN: convolutional neural network; XGB: extreme Gradient Boosting.

**Figure 4:**
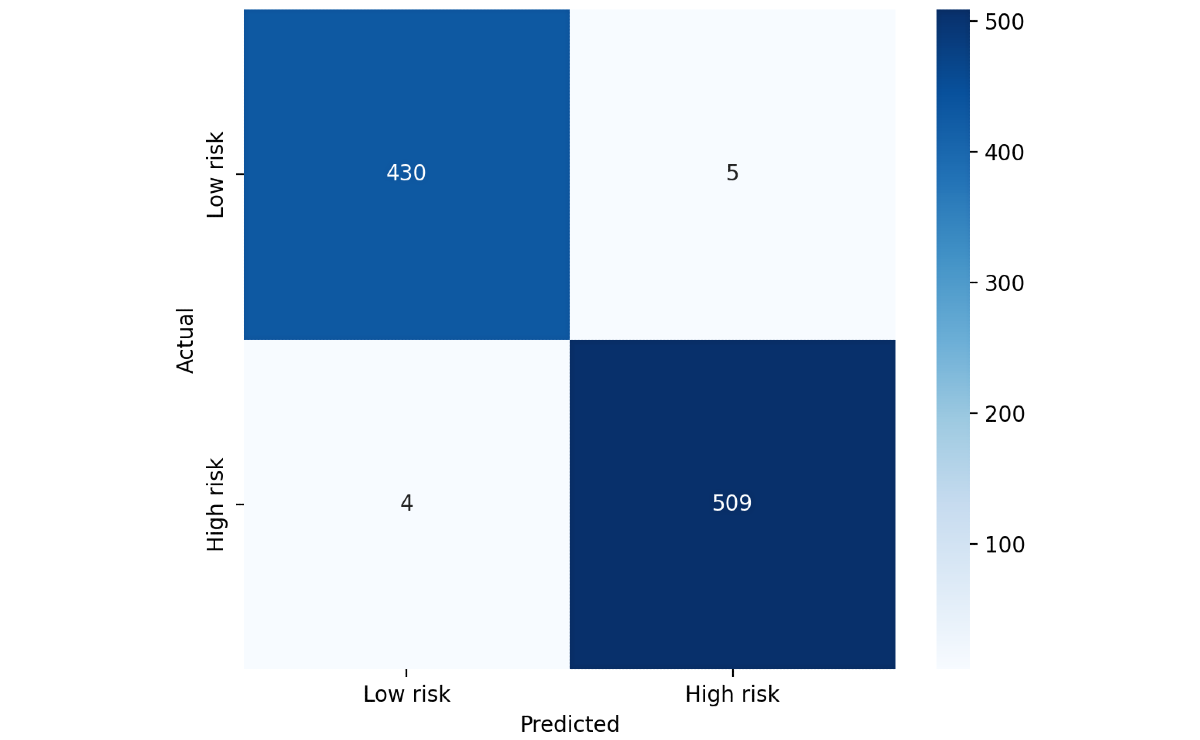
Confusion matrix of bidirectional encoder representations from transformers for biomedical text mining (BioBERT) using 2022-2023 train and 2024 test split.

**Figure 5:**
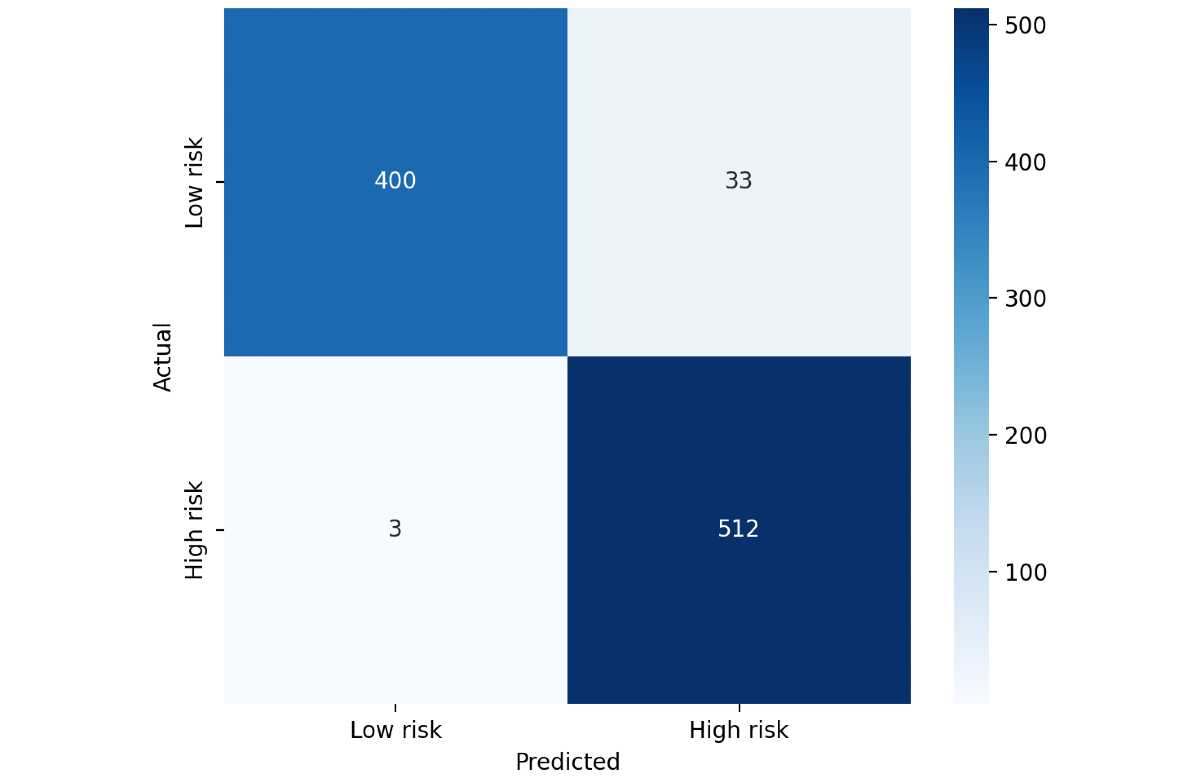
Confusion matrix of bidirectional encoder representations from transformers for biomedical text mining (BioBERT) using the traditional self-attention mechanism.

Table 9 provides the performance of a classification model in distinguishing between “Low Risk” and “High Risk” classes using a 5-fold CV score. The model performs exceptionally well for both categories, achieving high precision, recall, and *F*_1_-scores. For “Low Risk,” precision is 0.98, recall is 0.99, and the *F*_1_-score is 0.99, based on 1921 samples. Similarly, for “High Risk,” precision is 0.99, recall is 0.99, and the *F*_1_-score is 0.99, with 2601 samples. These results indicate a well-balanced model that accurately identifies both risk levels, with minimal misclassification. The confusion matrix for the proposed model based on CV is provided in Figure 3 (A, B, and C), providing a detailed view of true positives, true negatives, false positives, and false negatives. Figure 4 provides the confusion matrix obtained from a train-test split, demonstrating the model’s performance on temporally separated, unseen data and highlighting its real-world generalization capability. Finally, Figure 5 shows the confusion matrix of our proposed BioBERT model with the traditional self-attention mechanism.

**Table 9:** Class-wise evaluation scores of the fine-tuned biobert-base-cased-v1.1 model used in our proposed methodology.

Class	Precision	Recall	*F*_1_-score	Support
Low risk	0.98	0.99	0.99	1907
High risk	0.99	0.99	0.99	2568

### Statistical Analysis

To determine whether BioBERT’s superior performance over XGB is statistically significant across the 5 folds provided in Table 10 (F1 to F5), a paired t test was conducted using the *F*_1_-scores from each fold. The paired *t* test yielded a test statistic of *t*_4_=6.53 and a *P*=.003. Since the *P* value is well below the conventional threshold of .05, we reject the null hypothesis that there is no difference in performance between the 2 models. This result confirms that BioBERT’s higher performance is statistically significant and unlikely to be due to random chance. Therefore, based on the per-fold *F*_1_-scores, we conclude that BioBERT meaningfully and consistently outperforms XGB in this classification task.

**Table 10:** Comparison of accuracy across 5-fold cross-validation for bidirectional encoder representations from transformers for biomedical text mining (BioBERT) and XGB models.

Models	F1	F2	F3	F4	F5
XGB^a^	0.97	0.98	0.97	0.96	0.98
BioBERT^b^	0.99	0.99	0.99	0.98	0.99

^a^XGB: **e**xtreme Gradient Boosting.

^b^BioBERT: bidirectional encoder representations from transformers for biomedical text mining.

## Discussion

### Principal Findings

The results of this study underscore the significant potential of using advanced NLP techniques—particularly transformer-based models enhanced with custom attention mechanisms—for identifying opioid overdose risk levels from social media data. The exceptional performance of the proposed BioBERT-attention model, achieving an average CV score of 0.99, demonstrates its strong capability to capture nuanced semantic signals within Reddit posts that indicate high- or low-risk opioid use. This level of accuracy notably surpasses that of traditional and deep learning baselines, including the widely used XGB model, which, despite its robustness, achieved a lower score of 0.97. The statistically significant improvement, confirmed through a paired *t* test, further validates the effectiveness and reliability of the BioBERT-attention framework, affirming that the enhancements introduced by the custom attention layer contribute meaningful improvements rather than random performance fluctuations.

A key factor in this success is the domain-specific nature of BioBERT, which is pretrained on biomedical text and thus well-suited for understanding the health-related language and medical terminology frequently used in user-generated content discussing opioid use. The addition of a custom attention mechanism further allowed the model to emphasize contextually important cues—such as symptom descriptions, drug names, dosages, and subjective experiences—that are critical for determining the associated risk level. This tailored architecture addresses the shortcomings of generic models that may overlook such task-specific signals.

Moreover, the manual annotation of the dataset, guided by clearly defined labeling criteria, provided a strong foundation for supervised learning by ensuring consistency and high-quality ground truth. This highlights the value of combining expert-driven dataset curation with advanced model architectures in health-related NLP tasks. While the model shows excellent performance on the curated Reddit dataset, future research should explore its generalizability to other social media platforms and real-time deployment settings, where informal language, slang, or multilingual code-switching might pose additional challenges.

Overall, this study contributes a novel and effective approach to automated opioid risk detection by leveraging the rich, self-reported data available on Reddit. The proposed BioBERT-attention model not only achieves state-of-the-art performance but also presents a scalable framework that can be adapted to other substance abuse contexts, potentially transforming how public health agencies monitor and respond to emerging drug use trends in real time.

### Limitations

Despite the promising results demonstrated by our fine-tuned BioBERT model in detecting high-risk opioid substance use from Reddit data, several limitations must be acknowledged. First, our dataset is limited to Reddit posts, which may not capture the full diversity of opioid misuse discussions across different social media platforms or real-world contexts. The language, slang, and user demographics on Reddit may differ significantly from other online or offline populations, potentially limiting the generalizability of our findings. Second, although the dataset was manually annotated with detailed guidelines to ensure robustness, the subjective nature of annotation may introduce biases or inconsistencies. Additionally, some key contextual information, such as user history, temporal trends, or multimodal content (images and videos), was not considered, which could provide richer signals for more accurate risk detection. Third, the study focuses on supervised learning models with standard fine-tuning and does not explore more advanced domain adaptation techniques or novel architectures that might improve performance further, especially in handling imbalanced or noisy social media data.

Finally, the current evaluation is limited to computational experiments and statistical testing on CV folds, without real-world clinical validation or deployment. Future work should aim to validate these models in practical health care or intervention settings to assess their true impact and reliability in supporting harm reduction efforts.

### Conclusion and Future Work

The findings of our study underscore the significant potential of social media data in identifying opioid misuse patterns and assessing overdose risks. By creating a manually annotated dataset from Reddit, we have developed a valuable resource for categorizing opioids into high- and low-risk groups based on real user experiences. Our carefully crafted annotation guidelines ensure both accuracy and consistency, providing a solid foundation for future research in this area. Moreover, our experimental analysis, using 5-fold CV, demonstrated that transfer learning models using a custom attention mechanism significantly outperform traditional machine learning models such as XGB, achieving a performance improvement of 2.06%. This reinforces the power of AI-driven approaches in detecting opioid-related risks early, facilitating timely interventions and more effective harm reduction strategies. Ultimately, our work contributes to ongoing efforts to combat the opioid crisis by leveraging automation and real-world social media insights to support public health initiatives.

While our model has demonstrated strong performance using Reddit data with 5-fold CV, we acknowledge the importance of further evaluation to ensure broader robustness and generalizability. In future work, we plan to extend our dataset by incorporating opioid-related discussions from additional social media platforms such as Twitter and Facebook. This platform expansion will allow us to test the model’s adaptability and address platform shift challenges, ultimately improving cross-platform generalization. Additionally, we recognize the value of temporal evaluation and intend to conduct time-based splits to assess the model’s ability to generalize over evolving opioid-related discourse, addressing temporal generalization concerns. We also plan to explore domain-adaptive pretraining techniques to better tailor transformer models to opioid-specific language and context, which is expected to further enhance domain-specific understanding and improve classification robustness.

By pursuing these directions, we aim to develop a more resilient and widely applicable opioid misuse detection framework that can support public health monitoring and intervention across diverse and dynamic social media environments.

## Appendix

Multimedia Appendix 1 Word cloud visualizing the most frequent terms extracted from the dataset.

Multimedia Appendix 2 Dataset statistics.

Multimedia Appendix 3 Dataset label distribution.

## Abbreviations

AdaBoost: adaptive boosting.
BiLSTM: bidirectional long short-term memory
BioBERT: bidirectional encoder representations from transformers for biomedical text mining
ELECTRA: Efficiently Learning an Encoder that Classifies Token Replacements Accurately
CDC: Centers for Disease Control and Prevention
CNN: convolutional neural network
CV: cross-validation
DT: decision tree
GloVe: Global Vectors for Word Representation
IDF: inverse document frequency
MNB: multinomial naive Bayes
NLP: natural language processing
TF: term frequency
TF-IDF: term frequency–inverse document frequency
XGB: extreme Gradient Boosting
XLM-RoBERTa: Cross-lingual Language Model–Robustly Optimized BERT Pretraining Approach

## Data Availability

The datasets generated or analyzed during this study are not publicly available but are available from the first author upon reasonable request.
