# Supplementary figures and images for: Automated Risk Assessment of Opioid Use: Analysis Using Pre-Trained Transformers on Social Media Data
Source: JMIR Infodemiology. 2026 Feb 19;6:e77783. doi: 10.2196/77783 (PMC13147923; doi:10.2196/77783)

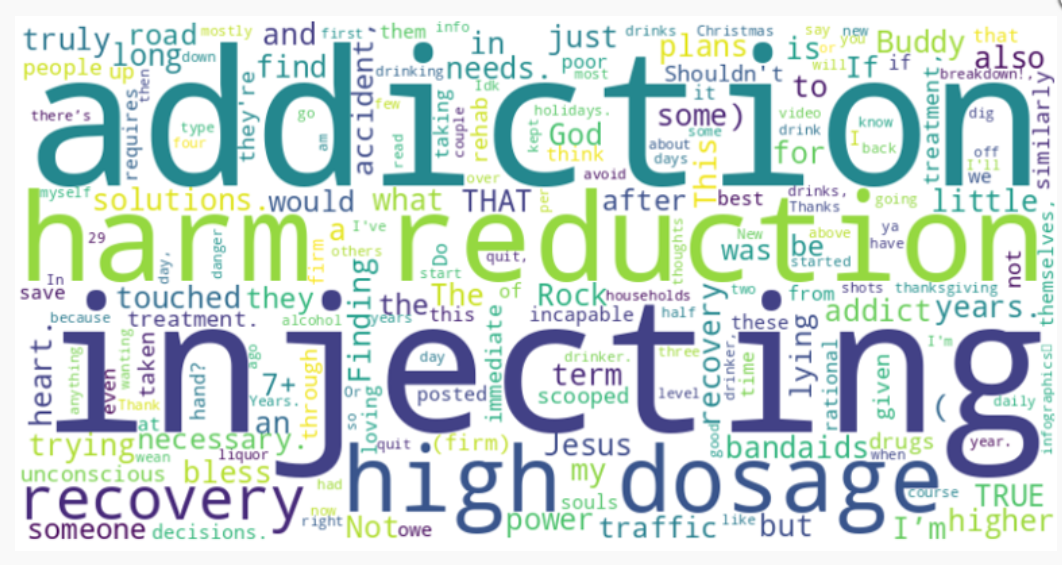

Supplement: Multimedia Appendix 1 [file infodemiology_v6i1e77783_app1.png]

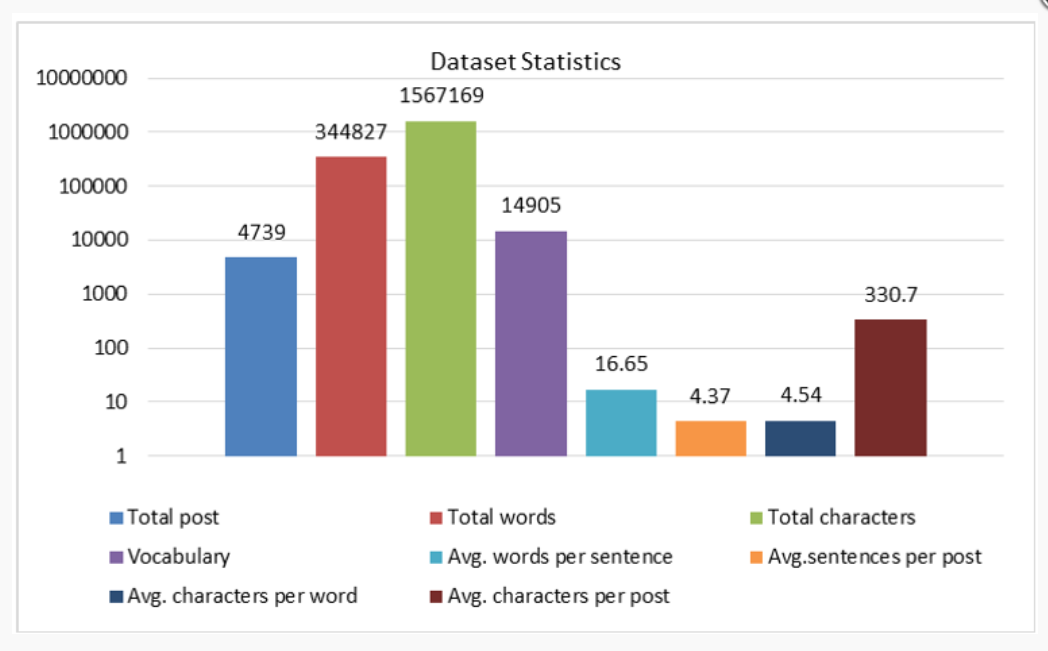

Supplement: Multimedia Appendix 2 [file infodemiology_v6i1e77783_app2.png]

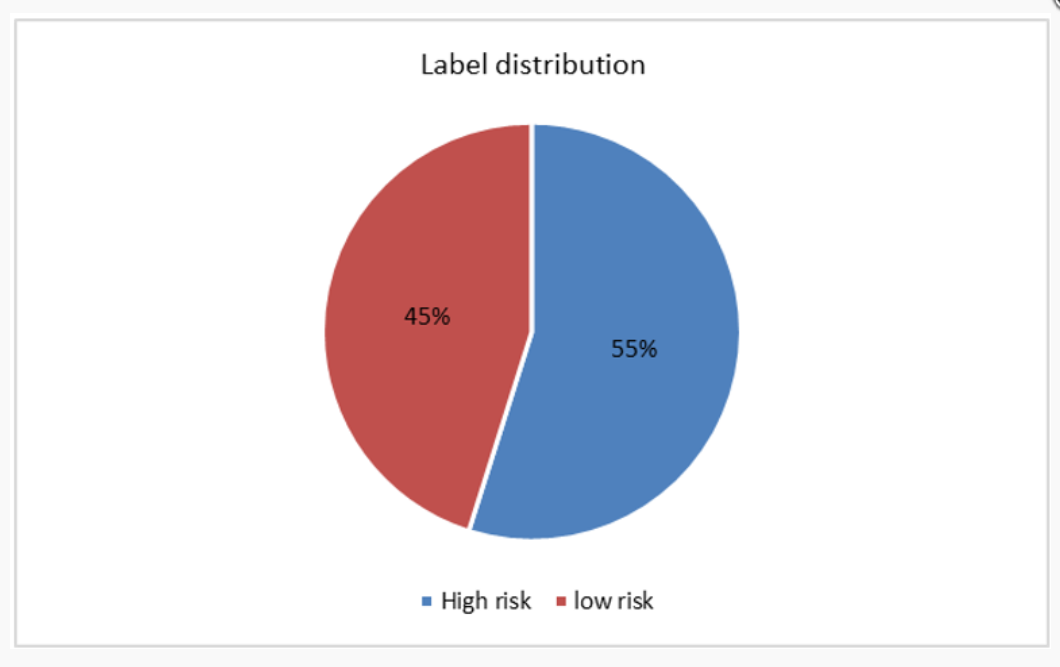

Supplement: Multimedia Appendix 3 [file infodemiology_v6i1e77783_app3.png]
